# Post-Translational Control of Nitrate Reductase Under Elevated CO_2_ in *Solanum lycopersicum*: Carbon–Nitrogen Signaling and Photosynthetic Acclimation

**DOI:** 10.3390/plants15172663

**Published:** 2026-08-31

**Authors:** Abhishek Sahoo, Mukesh Meena

**Affiliations:** Laboratory of Phytopathology and Microbial Biotechnology, Department of Botany, Mohanlal Sukhadia University, Udaipur 313001, Rajasthan, India; no1drabhisheksahoo@gmail.com

**Keywords:** nitrate reductase (NR), *Solanum lycopersicum*, elevated CO_2_ (eCO_2_), carbon–nitrogen interactions, nitrogen-use efficiency (NUE), climate change, omics approaches

## Abstract

Rising atmospheric CO_2_ is altering carbon–nitrogen interactions in C_3_ crops, with tomato (*Solanum lycopersicum* L.) showing enhanced carbon assimilation but frequently reduced nitrogen acquisition and assimilation. Nitrate reductase (NR), the rate-limiting enzyme in nitrate reduction, plays a central role by integrating nitrate assimilation with carbon metabolism and nitric oxide (NO) signaling. This review summarizes current knowledge of NR regulation in tomato under elevated CO_2_ (eCO_2_), focusing on post-translational mechanisms and their contribution to photosynthetic acclimation. Elevated CO_2_ modulates NR activity through interconnected changes in photorespiration, carbohydrate-mediated feedback, redox regulation, source–sink dynamics, and nitrogen availability. While eCO_2_ generally suppresses leaf nitrate assimilation by reducing photorespiratory support, root-zone CO_2_ enrichment can transiently stimulate root NR activity, highlighting tissue-specific regulation. Multi-omics studies further demonstrate extensive metabolic and molecular reprogramming affecting carbon skeleton supply, amino acid biosynthesis, and nitrogen assimilation. In addition, NR-dependent NO production links nitrogen metabolism with stomatal regulation through ABA-independent H_2_O_2_–NO signaling. Despite these advances, the roles of NR phosphorylation, 14-3-3 protein interactions, and redox-mediated regulation under eCO_2_ remain poorly understood. Overall, NR functions as a key metabolic and signaling hub coordinating carbon and nitrogen metabolism under future climate conditions. Understanding these regulatory mechanisms will facilitate strategies to improve nitrogen-use efficiency, sustain photosynthesis, and enhance tomato productivity under elevated atmospheric CO_2_ while identifying priorities for future physiological, molecular, and multi-omics research.

## 1. Introduction

Rising atmospheric CO_2_ concentrations are driving substantial metabolic reprogramming in C_3_ crop species, including *Solanum lycopersicum* L., in which enhanced carbon assimilation often occurs at the expense of nitrogen metabolism [1,2]. Although elevated CO_2_ enhances photosynthesis, its long-term benefits are constrained by nitrogen availability. In temperate forest ecosystems, productivity gains declined from 24% to 9% over an 11-year period [3], whereas a long-term Free-Air CO_2_ Enrichment (FACE) study involving multiple grassland plant species showed that the stimulation of biomass production weakened after 4–6 years under ambient nitrogen availability [4]. Responses scale with nitrogen acquisition (r^2^ = 0.68) and are overestimated by up to 85% without nitrogen constraints [5,6]. Mycorrhizal type further modulates outcomes, with ~30% biomass increase in ectomycorrhizal plants versus ~0% in arbuscular mycorrhizal plants under low nitrogen [7]. Elevated CO_2_ conditions in the range of 700–800 ppm, compared with ambient levels of 360–400 ppm, consistently induce nitrogen dilution, where increased biomass accumulation outpaces nitrogen acquisition, leading to reduced tissue nitrogen concentration, impaired protein synthesis, and compromised crop nutritional quality [8,9]. This imbalance reflects a decoupling of carbon and nitrogen assimilation pathways, in which nitrogen uptake and reduction fail to keep pace with the acceleration of carbon fixation [10]. Consequently, reductions in leaf nitrogen content and photosynthetic nitrogen-use efficiency represent a critical limitation on sustained productivity under future climate scenarios [11,12].

Empirical studies on tomato demonstrate that the effects of elevated CO_2_ on nitrogen metabolism are strongly context-dependent, varying with temperature, nitrogen form, and exposure duration. Under elevated CO_2_ at 700 ppm combined with chronic warming at 37 °C, nitrogen uptake rates per unit root mass decline significantly for both nitrate (NO_3_^−^) and ammonium (NH_4_^+^), accompanied by reduced concentrations of nitrogen uptake proteins [13]. These inhibitory effects are further exacerbated under 33/28 °C and 38/33 °C (day/night) regimes with 400 vs. 700 ppm CO_2_ and 2 mM ^15^NO_3_^−^/^15^NH_4_^+^, where elevated CO_2_ combined with warming reduces nitrogen uptake, net translocation, and the organic N: total N ratio over ≥21 days [14]. In contrast, under moderate temperature conditions (24 °C/18 °C day/night) and prolonged exposure (4 weeks) at 400 vs. 800 ppm CO_2_, ammonium nutrition (15 mM NH_4_^+^) enhances amino acid synthesis, particularly glutamine (Gln) and asparagine (Asn), supported by induction of anaplerotic enzymes such as malate dehydrogenase, NADP-malic enzyme, and isocitrate dehydrogenase [15]. These findings indicate that elevated CO_2_ can either inhibit or stimulate nitrogen metabolism depending on the environmental context.

The form and concentration of nitrogen supply further modulate these responses. Across studies, nitrogen treatments ranged from 0.2 mM to 15 mM NO_3_^−^ or NH_4_^+^, with differential outcomes. Under nitrate nutrition, elevated CO_2_ consistently suppresses nitrate assimilation, primarily by reducing photorespiration. Short-term exposure (≥2.5 h) at 360 ppm vs. 700 ppm CO_2_ and 25 °C/18 °C (day/night) decreases nitrate assimilation, as photorespiration under ambient conditions provides reductants necessary for nitrate reduction [16]. These differences are abolished under elevated CO_2_ or low oxygen (2% O_2_), confirming the dependence of nitrate assimilation on photorespiratory flux. In contrast, ammonium nutrition under elevated CO_2_ enhances carbon skeleton availability and promotes amino acid biosynthesis, facilitated by increased carbohydrate pools and TCA cycle activity at 400 vs. 800 ppm CO_2_ with 5–15 mM NH_4_^+^ [15,17]. This nitrogen source-specific response highlights the central role of carbon metabolism in regulating nitrogen assimilation.

Nitrate reductase (NR) serves as the primary rate-limiting gateway for nitrogen entry into cellular metabolism, catalyzing the reduction of NO_3_^−^ to NO_2_^−^ and occupying a central position in carbon–nitrogen homeostasis [18,19,20]. In tomato, NR activity is tightly coupled to photosynthetic electron transport and carbon skeleton availability, linking nitrogen assimilation with cellular energy status [21]. Notably, NR regulation is predominantly post-translational, involving rapid and reversible phosphorylation–dephosphorylation cycles and binding of 14-3-3 proteins, which modulate enzyme activity independently of protein synthesis [18,22,23]. These regulatory mechanisms enable dynamic adjustment of nitrogen assimilation in response to fluctuating environmental conditions, particularly changes in carbon availability under elevated CO_2_.

Despite its central role, direct evidence for post-translational regulation of NR under elevated CO_2_ remains limited. Viktor & Cramer [24] observed increased root NR activity under elevated CO_2_, with effects restricted to roots and not leaves, suggesting tissue-specific regulation. However, the underlying molecular mechanisms were not characterized. This lack of mechanistic resolution represents a significant gap in current understanding.

Temporal dynamics further complicate interpretation. Studies ranged from short-term exposure (≥2.5 h) to long-term experiments (4 weeks), with distinct outcomes. Short-term responses indicate immediate suppression of nitrate assimilation due to reduced photorespiration, whereas long-term exposure induces metabolic acclimation, including enhanced amino acid synthesis and activation of carbon-provision pathways [15,16]. Additionally, elevated CO_2_ stimulates photosynthesis by approximately 20% under ambient O_2_ (21%), but this effect is absent under low oxygen (2% O_2_), indicating that the benefits of CO_2_ enrichment are closely linked to photorespiratory processes. Collectively, elevated CO_2_ alters tomato nitrogen metabolism by altering photorespiration, carbon allocation, nitrogen assimilation, and NO/ROS signaling. However, the role of nitrate reductase (NR), particularly its post-translational regulation as a key integrator of carbon–nitrogen signaling, remains poorly understood.

This review therefore focuses on the post-translational control of nitrate reductase under elevated CO_2_ in tomato, aiming to elucidate how phosphorylation dynamics, 14-3-3 protein interactions, and redox regulation mediate the integration of carbon and nitrogen metabolism and drive photosynthetic acclimation. By synthesizing available physiological, biochemical, and molecular evidence, this work provides a framework for understanding nitrogen-use efficiency constraints and for identifying targets to improve crop performance under future elevated CO_2_ conditions.

## 2. Nitrogen Assimilation Pathway in Tomato

Nitrogen assimilation in *Solanum lycopersicum* depends on nitrate reductase (NR), the key regulatory enzyme that catalyzes the first committed step of nitrate reduction and links nitrogen metabolism with carbon availability and cellular energy status [19,25]. Because NR determines the entry of inorganic nitrogen into assimilatory pathways, its activity is tightly regulated in response to developmental cues, carbon status, and environmental conditions, making it a central target for post-translational regulation under elevated CO_2_ [26,27,28].

### 2.1. Nitrate Uptake via NRT Transporters

Nitrate acquisition in tomato roots is mediated by two kinetically distinct transporter systems: the low-affinity, high-capacity NRT1 family (LeNRT1.1, LeNRT1.2) and the high-affinity, low-capacity NRT2 family (LeNRT2.1, LeNRT2.2, LeNRT2.3) [29]. These transporter families correspond to the classical low-affinity transport system (LATS) and high-affinity transport system (HATS), enabling efficient nitrate uptake across a wide external concentration range (0.2–15 mM) [30,31]. Environmental stresses such as salinity and low temperature alter NRT transporter expression, thereby modifying nitrate uptake and its coordination with nitrogen assimilation [32,33].

Following uptake, nitrate is transported from roots to shoots, where nitrate reductase catalyzes the first committed step of nitrate assimilation. This root-to-shoot coordination links nitrate availability with photosynthetic carbon supply and provides the metabolic context for NR regulation under elevated CO_2_ [19,26].

### 2.2. Nitrate Reduction and Ammonium Assimilation

Nitrate reduction proceeds through two sequential enzymatic steps: cytosolic nitrate reductase (NR; EC 1.6.6.1) reduces nitrate to nitrite, followed by plastid-localized nitrite reductase (NiR; EC 1.6.6.4), which converts nitrite to ammonium [19,28]. NR is a homodimeric enzyme containing a molybdenum cofactor, heme, and FAD, enabling electron transfer from NADH to nitrate and representing the rate-limiting step in nitrogen assimilation [34,35].

**Figure 1:**
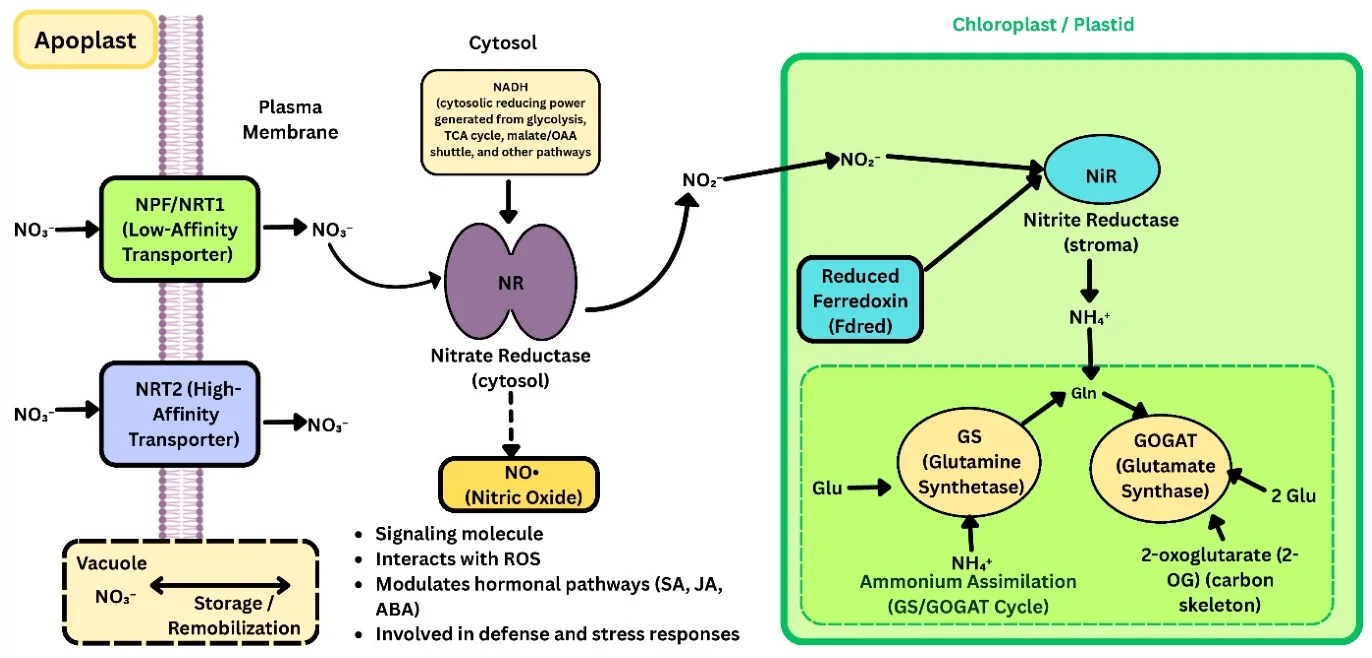
Cellular integration of the nitrogen assimilation pathway. Subcellular compartmentalization and sequential enzymatic steps of the nitrogen assimilation pathway in *Solanum lycopersicum*. Nitrate is taken up via NRT1 and NRT2 transporters and reduced to nitrite by cytosolic nitrate reductase (NR) using NADH. Nitrite is subsequently translocated to the plastid, where nitrite reductase (NiR) reduces it to ammonium for incorporation into amino acids via the GS/GOGAT cycle.

Increased PPF enhanced root NR activity, reflecting the close coupling between nitrate assimilation and photosynthetically derived carbon supply. The contrasting responses observed in leaves indicate tissue-specific regulation of NR activity and nitrogen assimilation within the plant [36]. Nitrogen assimilation in tomato is highly responsive to environmental stress and closely linked to carbon metabolism. Environmental stresses such as salinity alter tissue-specific NR activity and nitrogen assimilation, demonstrating the metabolic plasticity of tomato in maintaining nitrogen homeostasis (Figure 1). Nitrite reductase (NiR) exhibits a delayed response, decreasing by 25% in leaves and 40% in roots after 10 days of salt treatment. Following nitrate reduction, ammonium is assimilated through the GS/GOGAT cycle, which requires ATP, reducing power, and 2-oxoglutarate, thereby tightly coupling nitrogen assimilation with carbon metabolism [37]. Nitrogen form further influences this partitioning: ammonium nutrition favors root-based assimilation by increasing GS and GDH activities, whereas nitrate nutrition promotes shoot assimilation coordinated with photosynthetic carbon supply. These responses demonstrate the metabolic plasticity of tomato in maintaining nitrogen homeostasis under environmental stress.

### 2.3. GS/GOGAT Cycle and Amino Acid Biosynthesis

The GS/GOGAT cycle is the principal high-affinity pathway for ammonium assimilation, in which glutamine synthetase (GS) incorporates ammonium into glutamine and glutamate synthase (GOGAT) transfers the amide group to 2-oxoglutarate, generating glutamate for amino acid biosynthesis [38,39]. Multiple isoforms, including cytosolic GS1, chloroplastic GS2, ferredoxin-dependent GOGAT (Fd-GOGAT), and NADH-GOGAT, enable tissue-specific regulation of nitrogen metabolism [40]. Under 100 mM NaCl, tomato exhibits pronounced reprogramming of this pathway: leaf GS activity declines by 20–25% after 4 days, while Fd-GOGAT activity decreases by 38% within 1 day. In roots, GS activity initially increases after 7 days, but Fd-GOGAT falls to 10–25% of control levels, and NADH-GOGAT declines by 30–40% after 7–10 days, indicating severe impairment of primary ammonium assimilation [41]. These changes are associated with reduced carbon skeleton availability and promote a shift toward glutamate dehydrogenase (GDH)-mediated ammonium assimilation and detoxification.

During fruit development, nitrogen metabolism undergoes a similar transition from assimilation to amino acid interconversion. GS activity declines while NADH-GDH activity increases, accompanied by major changes in amino acid composition [42]. Glutamate accumulates to approximately 52% of total free amino acids in ripe Cherry tomato fruits, whereas γ-aminobutyric acid (GABA) predominates during early development (~60%) and glutamine reaches ~30% in mature green fruits. At the molecular level, tomato contains six *GS* genes, two *GOGAT* genes, and five *GDH* genes whose differential expression under stress and during development underpins these metabolic shifts [40,41]. Collectively, the GS/GOGAT–GDH balance represents a key regulatory mechanism that coordinates nitrogen assimilation, stress adaptation, and developmental nitrogen remobilization in tomato.

### 2.4. Nitrogen Allocation to Proteins, Chlorophyll, and Metabolites

In tomato, nitrate reductase (NR) plays a central role in determining nitrogen allocation by controlling the entry of inorganic nitrate into assimilatory pathways. Nitrogen assimilated through NR is subsequently partitioned among photosynthetic proteins, structural tissues, and developing fruits. During vegetative growth, a substantial proportion of nitrogen is invested in chloroplastic proteins such as Rubisco and chlorophyll-binding complexes, which account for much of the leaf nitrogen pool and determine photosynthetic capacity [43,44]. Under medium- and high-light conditions, shoot nitrogen concentrations remain relatively stable (55.4–59.3 mg g^−1^ DW), indicating efficient assimilation and utilization of absorbed nitrogen, whereas root nitrogen content can increase by up to 25.6%, reflecting enhanced nitrogen storage and transport capacity [43]. During reproductive development, nitrogen derived from NR-dependent assimilation is progressively remobilized from senescing leaves to developing fruits through protein degradation and phloem-mediated amino acid transport, supporting fruit growth and seed development [45]. Nitrogen availability strongly influences this process; reducing nitrate supply from 12 mM to 6–4 mM decreases yield by only ~7.5% but increases soluble sugars by 5–17% and decreases acidity by 10–16%, indicating a shift in carbon–nitrogen balance toward carbon-rich metabolites that enhance fruit quality [46]. These findings highlight the importance of NR-mediated nitrogen assimilation in regulating nitrogen partitioning, photosynthetic performance, nitrogen-use efficiency, and fruit quality in tomato.

## 3. Structure and Functional Biology of Nitrate Reductase in *Solanum lycopersicum*

### 3.1. Domain Organization (FAD, Heme, MoCo)

Nitrate reductase (NR) is a cytosolic homodimeric metallo-flavoenzyme (~100 kDa per monomer) that catalyzes the rate-limiting reduction of nitrate (NO_3_^−^) to nitrite (NO_2_^−^) in plants, fungi, and algae [47,48,49]. Each monomer exhibits a conserved modular architecture composed of three catalytic domains: an N-terminal flavin adenine dinucleotide (FAD)-binding domain, a central cytochrome b_5_-like heme domain, and a C-terminal molybdenum cofactor (MoCo)-binding domain. The enzyme is further organized into eight structural regions: (a) an N-terminal acidic region, (b) Mo-MPT catalytic domain, (c) interface domain, (d) Hinge 1 (harboring the regulatory phosphorylation site), (e) cytochrome b_5_ domain, (f) Hinge 2, (g) FAD domain, and (h) NAD(P)H-binding domain [50]. These domains are connected by flexible hinge regions that facilitate conformational rearrangements essential for catalysis and regulation. The FAD/NADH-binding region forms a two-lobed structure resolved at 2.5 Å, whereas the MoCo domain is deeply buried and stabilized by extensive hydrogen bonding networks [51,52].

The Mo center exhibits a pentacoordinate square pyramidal geometry, coordinated by two sulfur atoms of molybdopterin, one conserved cysteine residue, and two oxygen ligands [53,54,55]. The molybdenum must be chelated to a pterin to form the biologically active MoCo [55,56], with the pyranopterin dithiolene ligand stabilizing the metal center [57]. The dimerization domain adopts a Greek key motif, ensuring structural stability, and domain interfaces are reinforced by multiple interactions, including six salt bridges between the heme and reductase domains. An additional ~80-amino-acid acidic N-terminal extension is implicated in regulatory or structural roles [34].

### 3.2. Electron Transfer Mechanism

NR operates through a highly coordinated intramolecular electron transfer chain: NAD(P)H → FAD → heme → MoCo, culminating in nitrate reduction [58]. NAD(P)H donates two electrons to the FAD domain, which transfers them sequentially to the heme domain and subsequently to the MoCo catalytic center, where nitrate is reduced. Kinetic analyses demonstrate that electron transfer between domains occurs at comparable rates, with the heme-to-molybdenum step representing a critical regulatory point [50,59]. Under uninhibited conditions, the observed electron transfer rate (k_obs) is 310 s^−1^, with a nitrate binding affinity (K_d) of 131 μM. The catalytic turnover rate (k_cat) of the Mo domain is approximately 160 s^−1^, indicating high enzymatic efficiency [50,51]. Substrate affinity varies across systems, with K_m values ranging from 8–12 mM (spinach NR) to ~30 mM (yeast NR) [59].

Mechanistically, nitrate binds to the reduced Mo(IV) center, displacing a coordinated hydroxo/water ligand and oxidizing Mo(IV) to Mo(VI) [51,52]. The presence of four ordered water molecules in the active site stabilizes substrate binding and facilitates proton transfer. Specific residues, including arginine and tryptophan, contribute to substrate recognition within a defined binding pocket.

**Figure 2:**
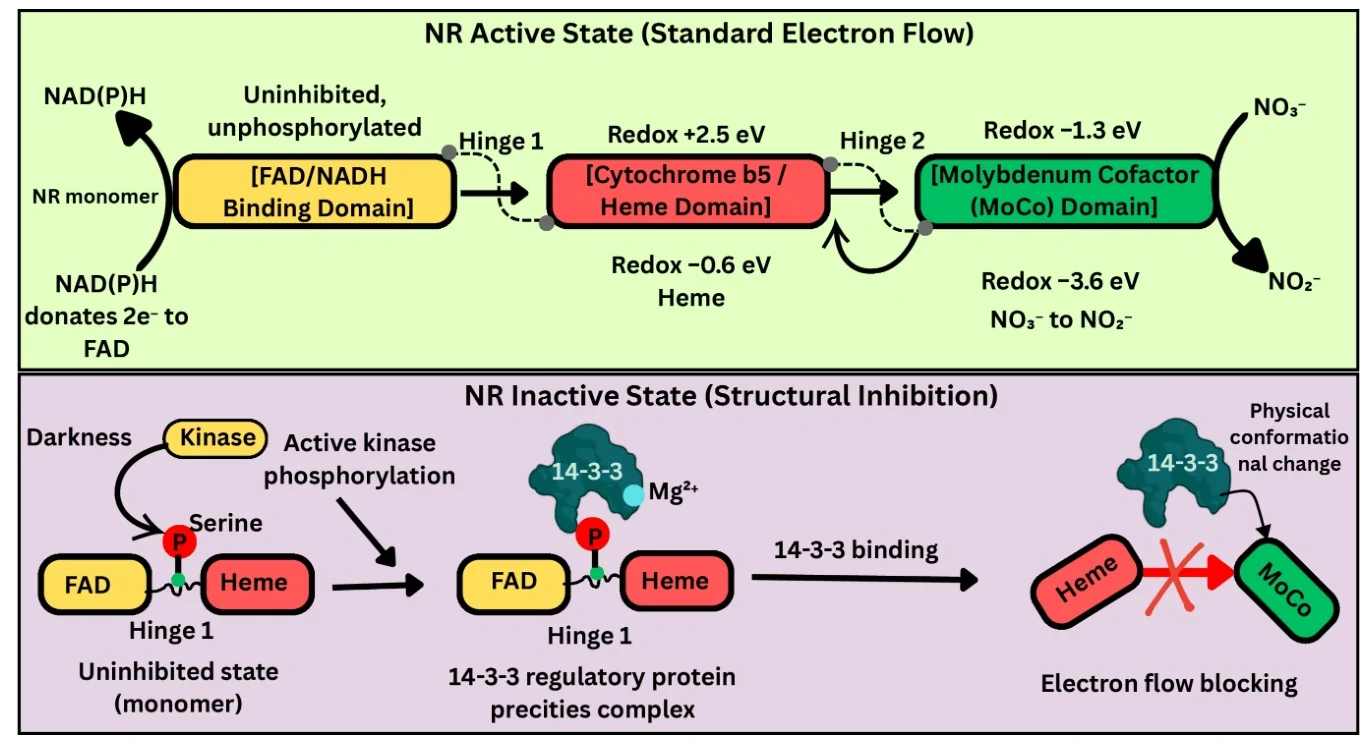
Structure, electron flow, and post-translational gating of nitrate reductase. Domain architecture and post-translational regulation of nitrate reductase (NR). In its active, unphosphorylated state, efficient intramolecular electron transfer proceeds from NAD(P)H through the FAD and heme domains to the active MoCo site. Phosphorylation at the conserved serine residue (Hinge 1) creates a binding motif for 14-3-3 proteins. Association of the 14-3-3 dimer induces a conformational shift that impedes electron transfer, reversibly inactivating the enzyme.

Electron transfer is strongly dependent on conformational flexibility. Phosphorylation at a conserved serine residue (e.g., Ser-534 in model plants) enables binding of 14-3-3 proteins, inducing a conformational transition from a “closed” (active) to an “open” (inactive) state. This increases the distance between redox centers and reduces electron transfer rates from 310 s^−1^ to ~35 s^−1^, without altering redox potentials [60]. Increased solution viscosity similarly reduces activity, confirming that domain mobility is essential. Product inhibition by nitrite (K_i ≈ 330 mM) provides an additional feedback mechanism preventing toxic accumulation (Figure 2).

### 3.3. NIA Gene Family and Isoforms in Tomato

In tomato, nitrate reductase is encoded by the NIA gene family, comprising at least two functional paralogs: SlNIA1 and SlNIA2 [61]. Among these, SlNIA2 is the dominant isoform, exhibiting higher transcript abundance and enzymatic activity and strong inducibility by nitrate. In contrast, SlNIA1 plays a comparatively minor role under standard conditions but is implicated in stress-related signaling functions [62,63].

Molecular characterization identified a tomato *nia* gene of approximately 5 kb, encoding a polypeptide of ~911 amino acids with three conserved introns [34]. Sequence alignment confirmed the presence of all three catalytic domains (MoCo, heme, FAD/NADH) and an N-terminal acidic extension. The promoter region contains a ~250 bp fragment with strong homology to tobacco *nia* genes, suggesting conserved regulatory mechanisms. Comparative analysis with solanaceous species reveals high conservation. In tobacco, two *nia* genes (*nia*-1: 13.5 kbp; *nia*-2: 12.6 kbp) encode proteins of ~904 amino acids, with identical intron positions and conserved catalytic domains [64]. Despite this conservation, the full composition and specialization of *NIA* genes in tomato remain unresolved, representing a key knowledge gap.

### 3.4. Tissue-Specific Expression and Activity Patterns

NR expression in tomato is strongly tissue-specific and regulated by environmental and metabolic cues. Highest expression and activity of SlNIA2 occur in mature leaves, reflecting the dependence of nitrate reduction on photosynthetic energy supply [47]. Roots and young leaves exhibit lower NR expression levels, while fruits show low but detectable NR expression during early development, which declines during ripening [65]. Although quantitative tissue-level datasets are limited, physiological evidence supports this distribution. NR-deficient mutants in related species exhibit chlorotic phenotypes, indicating the essential role of NR in leaf nitrogen metabolism [66]. Periclinal chimera studies further demonstrate that NR activity in specific cell layers (L2) is critical for maintaining nitrogen homeostasis [67].

NR activity is regulated at multiple levels. Transcriptionally, nitrate acts as a strong inducer of NIA gene expression [68]. Light enhances both gene expression and enzymatic activity via photosynthetic electron transport [58], while carbon status modulates NR to coordinate nitrogen assimilation with carbohydrate availability [69]. Environmental cues such as blue and UV-B light can synergistically induce NR expression, contributing to secondary metabolism (e.g., anthocyanin accumulation in Aft tomato) [65]. Post-translational regulation provides rapid control. Phosphorylation and 14-3-3 protein binding reversibly inhibit NR activity, allowing dynamic adjustment to environmental changes [49,70]. Additionally, inorganic phosphate acts as an activator, linking NR activity to cellular nutrient status. Beyond nitrate assimilation, NR contributes to nitric oxide (NO) production by reducing nitrite to NO via a one-electron reaction, integrating nitrogen metabolism with signaling pathways regulating growth, stomatal function, and stress responses [48,71,72,73,74].

## 4. Regulation of Nitrate Reductase

### 4.1. Transcriptional Control

#### 4.1.1. Induction by Nitrate and Repression Under Nitrogen Limitation

Transcriptional regulation is the primary level of control of nitrate reductase (NR) in tomato and determines the overall capacity for nitrate assimilation by modulating *Nia* gene expression [75]. Nitrate acts as the principal environmental inducer of NR transcription by activating conserved cis-regulatory elements located within the upstream promoter region of the tomato *nia* gene. Functional analyses in transgenic *Nicotiana plumbaginifolia* demonstrated that approximately 3 kb of the tomato *nia* promoter retains responsiveness to nitrate, light, and circadian regulation, indicating evolutionary conservation of the regulatory architecture governing nitrate assimilation [21]. Upon nitrate replenishment to nitrogen-starved plants, rapid accumulation of *Nia* transcripts occurs, thereby increasing NR protein synthesis and nitrate reduction capacity [76]. NR transcription is tightly regulated by the circadian clock in tomato, with transcript abundance increasing during the night, peaking near dawn, and declining by more than 100-fold by the end of the photoperiod [77]. This anticipatory regulation synchronizes nitrate assimilation with the availability of photosynthetic reductant, thereby optimizing carbon–nitrogen coordination and energy use.

Under nitrogen-limiting conditions or during the accumulation of reduced nitrogen metabolites, NR transcription is strongly feedback-repressed. The introduction of a functional *N. plumbaginifolia nia* locus into tomato reduced endogenous tomato *nia* transcript accumulation, supporting the hypothesis that nitrate-derived metabolites negatively regulate NR gene expression through metabolic feedback mechanisms [21]. Amino acids such as glutamine and asparagine function as indicators of nitrogen sufficiency and suppress further *Nia* induction, thereby preventing excessive nitrate reduction when downstream assimilation capacity becomes saturated [78,79]. Recent evidence further implicates NODULE-INCEPTION-like proteins (NLPs) as positive regulators of nitrate-inducible expression, whereas LATERAL ORGAN BOUNDARIES DOMAIN transcription factors (LBD37–LBD39) may mediate negative feedback repression under conditions of elevated nitrogen metabolite accumulation [80]. HY5-dependent light signaling has additionally been proposed to contribute to light-responsive NR expression. Collectively, these mechanisms establish a dynamic transcriptional framework that aligns nitrate assimilation with nutrient availability, circadian timing, and metabolic demand.

#### 4.1.2. Regulation by Carbon Metabolites and Sugars

Carbon metabolites, particularly sucrose and sugar phosphates, play a dominant role in regulating NR transcription and can override nitrate-induced signals. Sustained *Nia* expression depends on adequate carbon availability, with transcript levels declining sharply when sugar concentrations fall below ~4 μmol hexose equivalents g^−1^ fresh weight, regardless of nitrate supply [75]. Conversely, sucrose supplementation restores *Nia* transcript abundance and NR activity, demonstrating that sugar-mediated signaling tightly coordinates nitrate assimilation with cellular carbon status [81,82]. Sugars coordinate NR expression and activity with carbon availability, thereby integrating carbon and nitrogen metabolism [25,83,84]. Accordingly, NR activity declines in tomato tissues with reduced photosynthetic capacity or excessive nitrogen accumulation, ensuring that nitrate reduction proceeds only when sufficient carbon resources are available to support downstream metabolism [26,85].

### 4.2. Post-Translational Regulation

#### 4.2.1. Phosphorylation and Dephosphorylation Cycles

While transcriptional regulation establishes NR abundance, post-translational modifications enable rapid, reversible modulation of enzyme activity in response to fluctuating environmental conditions. The central mechanism underlying this regulation involves reversible phosphorylation of a conserved serine residue within the hinge region of the NR protein [86,87]. In tobacco NR, this regulatory residue corresponds to Ser-521, while the homologous residue in *Arabidopsis* is Ser-534 [23]. Phosphorylation converts NR into an inactive form, whereas dephosphorylation restores catalytic competence, thereby creating a rapid molecular switch that links nitrate reduction to cellular energy status [18]. Phosphorylation rapidly inactivates NR in darkness or when photosynthetic electron transport is restricted, whereas illumination and sugar accumulation promote dephosphorylation and activation [88,89]. This reversible post-translational mechanism enables rapid regulation of NR activity independent of protein abundance and contributes to diurnal oscillations in nitrate assimilation. In tomato, transient shading or chilling similarly induces rapid NR inactivation, preventing excessive nitrate reduction and the accumulation of toxic nitrogen intermediates when reductant availability is limited [77].

#### 4.2.2. 14-3-3 Protein-Mediated Inhibition

Phosphorylation of NR generates a recognition site for 14-3-3 regulatory proteins, whose association mediates potent inhibition of enzyme activity [60,90]. Binding of 14-3-3 proteins to phosphorylated NR requires the presence of divalent cations such as Mg^2+^ or polyamines, which stabilise the inhibitory complex and facilitate conformational changes that impede electron transfer between the heme and molybdenum cofactor domains [23]. This inhibition effectively suppresses nitrate reduction during darkness or metabolic stress when photosynthetic carbon assimilation and reductant supply become limiting.

**Figure 3:**
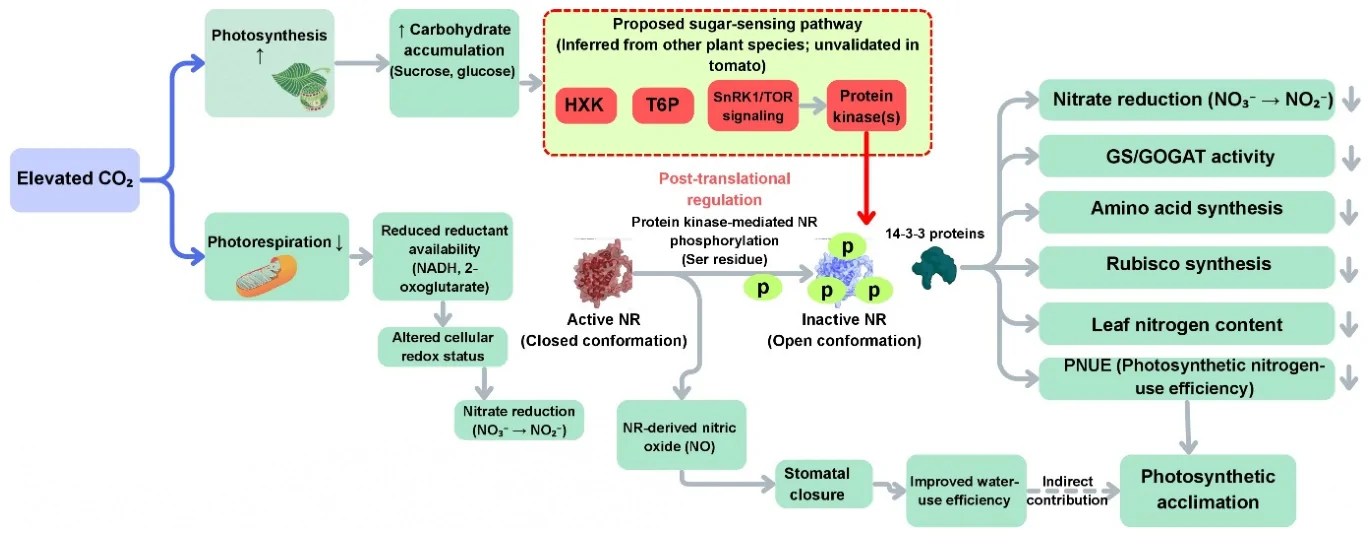
Proposed molecular mechanism of post-translational regulation of nitrate reductase (NR) under elevated CO_2_ in tomato. Elevated CO_2_ promotes carbohydrate accumulation and alters cellular redox status, leading to protein kinase-mediated NR phosphorylation and 14-3-3 protein binding, thereby reducing nitrate assimilation and downstream nitrogen metabolism. The proposed HXK–T6P–SnRK1/TOR signaling pathway is inferred from other plant species and remains unvalidated in tomato under elevated CO_2_. NR-derived nitric oxide (NO) promotes stomatal closure and indirectly contributes to photosynthetic acclimation.

The interaction between phosphorylated NR and 14-3-3 proteins additionally influences NR stability and degradation. MacKintosh & Meek [91] reported that extracellular sugars strongly influence 14-3-3 binding status and NR stability, linking carbon metabolism directly with enzyme turnover. Under normal conditions, NR protein exhibits a relatively short half-life of approximately 6 h, which decreases to nearly 2.5 h when plants are darkened during mid-day. Association with 14-3-3 proteins promotes destabilisation and proteolytic turnover of the inactive enzyme complex, whereas dissociation stabilises catalytically active NR [23]. Structural studies further indicate that the amino-terminal region contributes to proper inhibitory interactions and maintenance of enzyme stability, even though it is not strictly required for 14-3-3 binding itself [92]. These observations demonstrate that 14-3-3 proteins function not only as catalytic inhibitors but also as regulators of NR turnover dynamics. The proposed molecular framework linking elevated CO_2_-induced sugar signaling, NR phosphorylation, 14-3-3 protein binding, and NR-derived nitric oxide (NO) signaling is summarized in Figure 3.

#### 4.2.3. Redox and Light-Dependent Regulation

NR activity is intimately linked with chloroplast-derived redox signals and light-dependent metabolic regulation. Illumination promotes NR activation through coordinated dephosphorylation and redox-mediated modulation of enzyme thiol groups, thereby synchronising nitrate reduction with photosynthetic electron transport and reductant generation [18]. Thioredoxin-dependent signaling pathways communicate chloroplastic redox status directly to cytosolic NR, ensuring that nitrate assimilation proceeds only when sufficient reducing power and carbon skeletons are available [93,94]. Environmental stresses strongly influence NR through redox-sensitive post-translational regulation. In tomato, overnight chilling induces NR transcription but rapidly suppresses NR activity, thereby uncoupling carbon and nitrogen assimilation while preventing excessive nitrite and NO accumulation under reduced photosynthetic activity [77,95]. This effect is reversible, with circadian rhythms recovering after stress relief [96]. Similarly, changes in light intensity, oxygen availability, and transient shading rapidly modulate NR activity through post-translational mechanisms, preventing unnecessary nitrate reduction when carbon assimilation is limited [23].

### 4.3. Metabolic Feedback

#### 4.3.1. Amino Acid Feedback Inhibition

Metabolic feedback regulation provides an additional layer of control coordinating nitrate assimilation with downstream nitrogen metabolism [97]. Amino acids, particularly glutamine and asparagine, function as key indicators of nitrogen sufficiency and exert strong inhibitory effects on NR expression and activity [78]. Glutamine acts as a direct repressor of NR synthesis, whereas ammonia-mediated repression occurs indirectly through glutamine formation [98]. Such feedback inhibition prevents excessive nitrate reduction when amino acid pools become saturated and downstream assimilation demand declines.

Studies of deregulated NR lines revealed profound metabolic consequences when post-translational control is abolished. Lea et al. [99] demonstrated that glutamine and asparagine concentrations increased approximately 9-fold and 14-fold, respectively, in fully deregulated plants compared with wild-type controls. Additional amino acids, including isoleucine and threonine, accumulated substantially, whereas nitrate uptake rates remained largely unchanged. This indicates that NR regulation primarily affects nitrogen assimilation and metabolite partitioning rather than nitrate acquisition, highlighting its critical role in maintaining amino acid homeostasis and metabolic balance.

Amino acid-mediated repression appears to operate independently of hexokinase-dependent sugar signaling pathways, suggesting that nitrogen metabolites utilize distinct regulatory networks for controlling nitrate assimilation [100]. In root tissues, amino acids may also compete for transport pathways or exert antagonistic biochemical interactions that influence NR induction kinetics [101]. Secondary metabolites such as coumarin and cinnamic acid derivatives also inhibit NR induction, indicating that metabolic feedback regulation extends beyond primary nitrogen metabolism into broader defense-associated pathways [102]. Collectively, these interactions fine-tune nitrate assimilation according to cellular metabolic requirements and environmental conditions.

#### 4.3.2. Integration with Carbon and Energy Status

The integration of nitrogen assimilation with carbon and energy metabolism is fundamentally governed by the cellular C/N ratio. NR activity declines when carbon availability becomes insufficient relative to nitrogen assimilation demand, thereby preventing depletion of reductant pools and maintaining metabolic homeostasis [103]. Sugars function as dominant regulatory signals that can override nitrate-induced *Nia* expression when carbon resources become limiting [104]. When sugar concentrations fall below the threshold necessary for sustained NR transcription, nitrate assimilation is rapidly repressed despite high nitrate availability and low glutamine concentrations [75]. Conversely, elevated sugar availability supports *Nia* transcription, enhances NR activation state, and promotes nitrate assimilation by supplying both energy and carbon skeletons required for amino acid biosynthesis [79,100]. Sucrose supplementation can partially alleviate amino acid-mediated repression, demonstrating that carbon metabolites actively counterbalance inhibitory nitrogen signals [78].

Organic acids and photosynthetically derived carbohydrates further coordinate nitrate reduction with growth and developmental demands. Sugars and sugar phosphates regulate kinases and phosphatases that control NR activation state, thereby linking carbon metabolism directly to post-translational NR regulation [23]. Exogenous carbon compounds such as sucrose and glucose positively influence root growth, nitrogen uptake, and whole-plant C/N balance [105]. However, under carbon-limiting conditions or during environmental stress, transient uncoupling of carbon and nitrogen assimilation depletes reductant reserves and permits the accumulation of cytotoxic nitrogen-metabolism intermediates [96]. Collectively, these findings indicate that NR regulation in tomato is governed by a multi-layered network involving transcriptional control, post-translational modification, metabolite signaling, and carbon–nitrogen coordination. This regulatory framework ensures that nitrate assimilation remains tightly synchronized with cellular energy status, environmental conditions, and developmental demands while preventing the accumulation of toxic nitrogen intermediates.

## 5. Effects of Elevated CO_2_ on Nitrate Reductase Activity in *Solanum lycopersicum*

Elevated atmospheric CO_2_ profoundly modifies nitrate reductase (NR)-mediated nitrogen assimilation in *Solanum lycopersicum*, generating a physiological disequilibrium between accelerated carbon fixation and progressively constrained nitrogen metabolism [106,107]. Although elevated CO_2_ initially enhances photosynthesis, prolonged exposure often suppresses nitrate assimilation, reducing amino acid synthesis, protein accumulation, and nitrogen investment in photosynthetic machinery [108,109]. Nitrate reductase (NR) responses are highly context-dependent, varying with exposure duration, tissue type, genotype, nitrogen source, source–sink balance, and environmental conditions [110]. Current evidence suggests that elevated CO_2_ regulates NR through interconnected effects on photorespiration, redox status, carbohydrate signaling, and whole-plant carbon–nitrogen coordination.

### 5.1. Differential Regulation of Nitrate Reductase Activity Under Elevated CO_2_

Elevated CO_2_ exerts tissue-specific effects on nitrate reductase (NR) activity and nitrate assimilation. In tomato cv. F144, root-zone CO_2_ enrichment (5000 ppm) increased root NR activity by approximately 43%, from 11.9 ± 1.1 to 17.0 ± 0.6 µmol g^−1^ h^−1^, accompanied by transient stimulation of nitrate uptake and root growth during the first 6–12 h of exposure; however, these effects diminished with prolonged treatment, while leaf NR activity declined [24]. In contrast, elevated atmospheric CO_2_ suppresses leaf nitrate assimilation by reducing photorespiratory support for nitrate reduction despite increased carbon fixation [16]. Long-term exposure also disrupts the normal relationship between NR activity and leaf nitrogen concentration in tomato cultivars ‘Vendor’ and ‘Carmelo’, indicating fundamental alterations in nitrogen regulatory networks [111]. Similar reductions in NR activity and nitrogen assimilation have been reported in tobacco, wheat, and rice under elevated CO_2_, suggesting a conserved response across C_3_ species.

### 5.2. Photorespiratory Suppression and Redox Limitation

Suppression of photorespiration is widely considered a major contributor to reduced nitrate assimilation under elevated CO_2_ (eCO_2_). By increasing the CO_2_/O_2_ ratio at Rubisco, eCO_2_ reduces photorespiratory flux, disrupting the metabolic integration of carbon and nitrogen assimilation [107,112]. Reduced photorespiration limits the supply of reducing power, 2-oxoglutarate, and recycled ammonium required for NR activity and GS/GOGAT-mediated nitrogen assimilation [113,114]. Recent evidence further indicates that transporters such as NPF8.4 coordinate photorespiratory carbon flux with nitrogen status by regulating glycerate partitioning under nitrogen limitation, highlighting the dynamic linkage between photorespiration and nitrogen metabolism [115].

The inhibitory effects of eCO_2_ are generally stronger under nitrate than ammonium nutrition because nitrate assimilation requires additional reducing power and metabolic support from photorespiration [116]. Collectively, these findings indicate that suppression of photorespiration constrains nitrate assimilation by influencing redox balance, carbon skeleton availability, nitrogen recycling, transporter-mediated metabolic coordination, and carbon–nitrogen signaling.

### 5.3. Carbon Excess and Carbohydrate-Driven Feedback Inhibition

Elevated CO_2_ stimulates photosynthetic carbon fixation and promotes the accumulation of non-structural carbohydrates, particularly sucrose and starch, when sink demand or nitrogen availability limits their utilization [117,118]. These carbohydrates function not only as metabolic substrates but also as signaling molecules that repress nitrogen assimilation pathways, including nitrate reductase (NR), thereby altering carbon–nitrogen balance [114,119]. Competition for reducing power between carbon fixation and nitrate reduction may further constrain NR activity under elevated CO_2_, particularly when sink strength is limited [120,121]. Collectively, carbohydrate accumulation contributes to reduced NR activity under elevated CO_2_, whereas the underlying signaling mechanisms coordinating carbon–nitrogen crosstalk are discussed in the context of nitrate reductase in carbon–nitrogen crosstalk in tomato.

### 5.4. Acclimation Dynamics and Whole-Plant Nitrogen Redistribution

The effects of elevated CO_2_ on NR activity are strongly time-dependent [14]. Short-term exposure can transiently stimulate nitrate uptake, root metabolism, and NR activity through increased carbon availability and growth demand, whereas prolonged exposure promotes metabolic acclimation characterized by feedback inhibition of nitrate assimilation [122,123]. Long-term responses involve transcriptional reprogramming, altered source–sink relationships, reduced nitrate transport and shoot nitrogen allocation, and progressive nitrogen dilution [124,125].

## 6. Nitrate Reductase in Carbon–Nitrogen Crosstalk in Tomato

### 6.1. Sugars as Signaling Molecules Regulating NR Activity

Under elevated CO_2_ conditions, hexose sugars, particularly glucose and sucrose, function as major signaling molecules that regulate nitrate reductase (NR) activity and coordinate carbon–nitrogen interactions in tomato through both hexokinase-dependent and independent pathways [126,127,128]. Hexokinase (HXK) acts as a glucose sensor, modulating nitrogen assimilation gene expression and protein stability, thereby linking photosynthetic carbon fixation to nitrogen metabolism [129]. Elevated CO_2_-grown tomato fruits accumulated significantly higher glucose and fructose concentrations compared with ambient conditions, while sucrose concentrations remained largely unchanged, with fructose becoming the predominant soluble sugar during fruit maturation [130]. The same study further demonstrated significantly higher soluble acid invertase and sucrose synthase activities under elevated CO_2_, indicating enhanced photosynthate metabolism and carbohydrate translocation. Studies in tomato demonstrated that sucrose and glucose mimic elevated CO_2_-induced repression of photosynthetic gene expression, whereas acetate and sorbitol do not, indicating that specific sugars act as signaling molecules driving photosynthetic acclimation [131]. Trehalose-6-phosphate (T6P) has been proposed as an important regulator linking sucrose status with TOR and SnRK1 signaling and, consequently, nitrate assimilation; however, its role under elevated CO_2_ has not yet been directly examined in tomato [132,133].

### 6.2. Adjustment of the C:N Ratio Under Elevated CO_2_

Elevated atmospheric CO_2_ substantially modifies carbon–nitrogen (C:N) balance in tomato by increasing photosynthetic carbon fixation and altering nitrogen assimilation pathways [134,135]. Nitrogen source strongly influenced metabolic adjustment patterns under elevated CO_2_ [136]. Tomato studies show that carbohydrate accumulation under elevated CO_2_ represses photosynthesis-related genes and likely coordinates nitrate assimilation with sink utilization and carbon skeleton availability [131,137].

### 6.3. NR as a Metabolic Checkpoint Linking Carbon Availability and Nitrogen Assimilation

**Figure 4:**
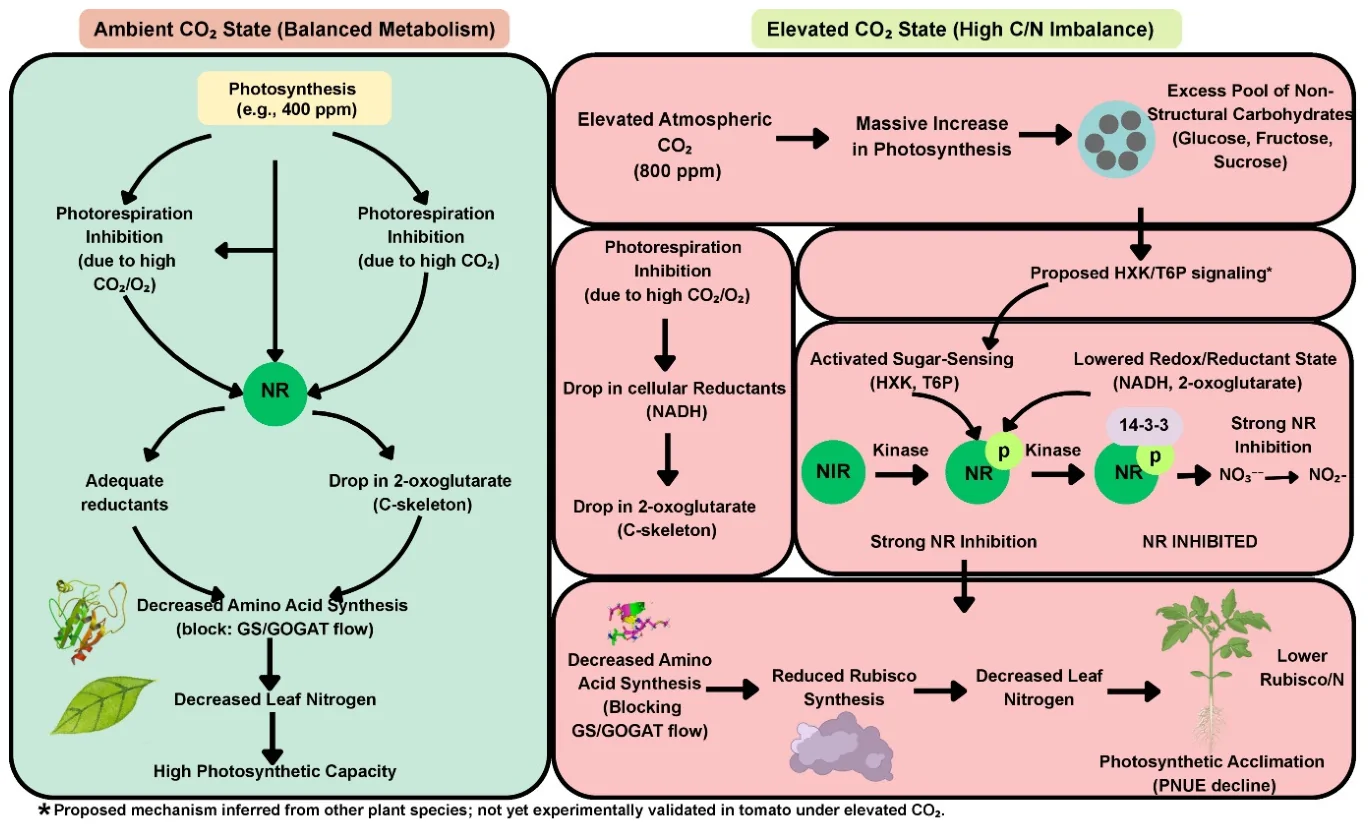
Systemic carbon–nitrogen crosstalk under elevated CO_2_. Proposed conceptual model illustrating the post-translational regulation of nitrate reductase (NR) and its role in carbon–nitrogen interactions under elevated CO_2_ in tomato. Elevated CO_2_ enhances carbon fixation while suppressing photorespiration, leading to carbohydrate accumulation, altered redox status, and reduced availability of reductants and carbon skeletons for nitrogen assimilation. These changes promote post-translational inhibition of NR, restricting nitrogen flux through the GS/GOGAT cycle and reducing photosynthetic protein synthesis, ultimately contributing to photosynthetic acclimation. Sugar-sensing pathways (e.g., hexokinase and trehalose-6-phosphate [T6P]) regulating NR phosphorylation and 14-3-3-mediated inhibition are proposed based on evidence from other plant species and remain unvalidated in tomato under elevated CO_2_.

Nitrate reductase serves as a central metabolic checkpoint, linking carbon availability to nitrogen assimilation through integrated transcriptional and post-translational regulation [138]. The checkpoint function of NR depends on reversible phosphorylation, 14-3-3 protein binding, and carbohydrate-responsive signaling pathways that rapidly adjust nitrate reduction in response to carbon status [60,91]. Under elevated CO_2_, enhanced carbohydrate accumulation and altered sink–source balance are proposed to increase NR phosphorylation and inhibition, thereby preventing excessive nitrate reduction when carbon utilization becomes limiting (Figure 4). Although direct measurements of NR activity under elevated CO_2_ in tomato remain limited, indirect evidence supports carbon-mediated regulation of nitrogen assimilation. Elevated CO_2_ increases glucose and fructose accumulation, modifies invertase and sucrose synthase activities, and alters TCA cycle metabolism, indicating substantial reprogramming of carbon–nitrogen interactions [17,130,139]. Additionally, enhanced photorespiratory serine production under NH_4_^+^ nutrition suggests compensatory metabolic mechanisms that support nitrogen incorporation during carbon–nitrogen imbalance [139].

The interaction among carbohydrate accumulation, carbon skeleton availability, and nitrogen assimilation likely involves coordinated regulation via glucose, sucrose, T6P, TOR, and SnRK1 signaling networks [140]. Nevertheless, direct measurements of NR activity, phosphorylation status, and T6P-mediated regulation under elevated CO_2_ conditions in tomato remain absent, highlighting a critical research gap in understanding NR-mediated carbon–nitrogen crosstalk under future climate scenarios [17,131,139].

## 7. Omics Approaches to Decipher Nitrate Reductase (NR) Regulation Under Elevated CO_2_

Understanding nitrate reductase (NR) regulation under elevated atmospheric CO_2_ is central to elucidating how tomato plants coordinate carbon (C) and nitrogen (N) metabolism during climate change [14]. Although direct studies on nitrate reductase (NR) regulation are limited, evidence from transcriptomics, translatomics, metabolomics, proteomics, physiological analyses, and gene-silencing studies reveals a complex network linking photosynthetic acclimation, stomatal signaling, carbohydrate metabolism, photorespiration, and nitrogen assimilation under elevated CO_2_ (typically 700–800 ppm versus 380–400 ppm ambient CO_2_). Responses vary according to genotype, tissue, nitrogen source, developmental stage, and environmental conditions.

### 7.1. Transcriptomic Insights into NR Regulation Under Elevated CO_2_

Transcriptomics has been the most widely employed omics approach for investigating tomato responses to elevated CO_2_. Shi et al. [141] provided direct evidence for NR regulation under elevated CO_2_ using qPCR in the Ailsa Craig variety of tomato and the ABA-deficient mutant notabilis exposed to 800 vs. 380 ppm CO_2_. In the second fully expanded leaves, elevated CO_2_ induced a 1–4-fold increase in OST1, RBOH1, NR, and SLAC1 transcript abundance and triggered stomatal closure within 30 min. Elevated CO_2_ also increased NR activity and activation state, indicating both transcriptional and post-translational regulation. VIGS experiments demonstrated that NR acts downstream of RBOH1-dependent H_2_O_2_ signaling to mediate NO production during stomatal closure, establishing NR as a key component of the ABA-independent H_2_O_2_–NO signaling pathway induced by elevated CO_2_.

Gray et al. [142] revealed enrichment of glycolysis- and TCA-cycle pathways and extensive translational regulation, suggesting coordinated carbon skeleton provision for nitrogen assimilation (Table 1). Analysis of leaves and roots collected at 28–35 DAP showed that elevated CO_2_ increased photosynthetic carbon assimilation (F_1,92_ = 24.5, *p* < 0.05) while decreasing stomatal conductance (F_1,92_ = 23.9, *p* < 0.05). Enrichment of glycolysis- and TCA cycle-related genes, together with organ-specific root transcriptomic responses, suggested enhanced carbon skeleton production and coordinated regulation of nitrogen uptake and assimilation under elevated CO_2_.

Transcriptomic studies indicate that elevated CO_2_ influences NR regulation both directly and indirectly through broader carbon–nitrogen metabolic networks. In *S. lycopersicum*, [139] showed that exposure to 400 and 800 ppm CO_2_ under nitrate (NO_3_^−^) or ammonium (NH_4_^+^) nutrition altered expression of genes involved in carbonic anhydrase activity, photorespiration, glyoxylate-cycle metabolism, and carbon-provision pathways, suggesting enhanced carbon skeleton availability to support nitrogen assimilation. Likewise, Van Oosten et al. [131] reported that tomato leaves exposed to elevated CO_2_ for up to 9 days exhibited reduced transcript abundance of *cab-7*, *cab-3C*, and *Rca*, whereas *psaA–psaB*, *psbA*, and glycolate oxidase transcripts remained unchanged. Elevated CO_2_ also induced expression of the B subunit of ADP-glucose pyrophosphorylase within 1 day, indicating that carbohydrate accumulation drives transcriptional reprogramming. Together, these studies suggest that elevated CO_2_ reshapes carbon metabolism and sugar signaling pathways that may indirectly regulate NR expression and nitrogen assimilation [131,139].

### 7.2. Translatomic Regulation and Post-Transcriptional Control of Nitrogen Metabolism

One of the most significant methodological advances in elevated CO_2_ research was the implementation of TRAP-Seq by Gray et al. [142]. Unlike conventional transcriptomics, TRAP-Seq captures ribosome-associated mRNAs and therefore provides insight into translational regulation. Despite substantial physiological responses to elevated CO_2_, many metabolite changes occurred without corresponding transcriptional alterations, indicating extensive post-transcriptional regulation. Differential polysome occupancy was observed for transcripts involved in transcriptional initiation and regulatory functions. Members of the Mediator complex, including MED11, MED18, and MED19B, emerged as previously unrecognized components of CO_2_-responsive regulatory networks.

### 7.3. Metabolomic Evidence Linking Carbon Status and NR Function

Metabolomics has provided critical insights into the metabolic context within which NR operates under elevated CO_2_. Van Oosten et al. [131] reported substantial accumulation of glucose and fructose in leaves exposed to elevated CO_2_. Exogenous application of glucose or sucrose reproduced the transcriptional responses observed under elevated CO_2_, whereas acetate and sorbitol did not. Furthermore, gene expression responses were amplified when leaves were detached from the source–sink networks. These observations demonstrated that carbohydrate accumulation functions as a signaling mechanism linking carbon availability to nuclear gene regulation. Because NR activity is tightly coordinated with carbon status, these findings suggest that sugar-mediated signaling may indirectly regulate NR expression and activity.

Using integrated metabolomics and transcriptomics, Gray et al. [142] detected significant changes in amino acid metabolism. Elevated CO_2_ altered concentrations of glutamic acid (logFC = 0.97), ornithine (logFC = 1.44), and aspartic acid (logFC = 1.56) in shoots, while phenylalanine (logFC = 0.69) increased and fumaric acid (logFC = −1.11) decreased in roots. Metabolomic analyses consistently indicate substantial reprogramming of amino acid and organic acid metabolism under elevated CO_2_, supporting altered carbon–nitrogen coordination (Table 2).

Similarly, Vega-Mas et al. [17] investigated the tomato cultivar Agora Hybrid F1 under ambient (400 ppm) and elevated (800 ppm) CO_2_ supplied with either nitrate or ammonium at concentrations ranging from 5–15 mM. Metabolomic analyses revealed substantial reorganization of carbon and nitrogen metabolism. Elevated CO_2_ altered the coordination between nitrogen-assimilating enzymes and carbon-anaplerotic pathways and abolished the induction of TCA-cycle anaplerotic enzymes normally observed under ambient CO_2_ during non-toxic ammonium nutrition. These findings indicate that elevated CO_2_ fundamentally reshapes the metabolic framework supporting NR-dependent nitrogen assimilation.

### 7.4. Proteomic Perspectives on Nitrogen Assimilation Under Elevated CO_2_

Proteomic investigations remain relatively limited but provide valuable evidence concerning downstream consequences of NR regulation. Jayawardena et al. [13] employed root proteomics to examine tomato responses to elevated CO_2_ (700 ppm) and different nitrogen sources (NO_3_^−^ and NH_4_^+^). Elevated CO_2_, particularly when combined with chronic warming, resulted in nitrogen dilution, reduced nitrogen uptake rates, and decreased total protein concentration. These findings indicate suppression of overall nitrogen assimilation despite enhanced carbon availability, highlighting environmental constraints on NR-mediated nitrogen metabolism. Proteomic analyses by Vega-Mas et al. [17] further demonstrated that elevated CO_2_ alters the abundance and activity of proteins involved in ammonium assimilation and carbon metabolism. The observed metabolic adjustments suggest that elevated CO_2_ influences nitrogen-assimilation efficiency not only through transcriptional regulation but also through protein-level reorganization of metabolic pathways.

### 7.5. NR Within Elevated CO_2_-Induced Signaling Networks

The strongest mechanistic evidence for NR regulation under elevated CO_2_ comes from the signaling framework established by Shi et al. [141]. Elevated CO_2_ stimulated RBOH1-dependent H_2_O_2_ production, which subsequently activated NR-dependent NO synthesis. Nitric oxide then promoted stomatal closure by regulating *SLAC1*, establishing a coordinated signaling pathway independent of ABA biosynthesis. The sequence of events was supported through pharmacological treatments, confocal microscopy, transmission electron microscopy, VIGS-mediated gene silencing, and real-time visualization of H_2_O_2_ and NO production. The study further identified reciprocal interactions between H_2_O_2_ and NO, suggesting the existence of a feedback-regulated signaling module. These findings substantially revised previous models by demonstrating that elevated CO_2_-induced stomatal closure can occur independently of ABA accumulation and instead relies on NR-mediated NO production.

## 8. Coupling with Photosynthetic Acclimation

### 8.1. Nitrogen Allocation to Rubisco and Chlorophyll

Nitrogen partitioning within leaves balances investment between Rubisco, the major nitrogen sink, and light-harvesting components such as chlorophyll-binding and thylakoid proteins [132,147]. In tomato, this allocation is highly plastic, with young sun-exposed leaves favoring Rubisco accumulation and older or shaded leaves reallocating nitrogen to other tissues [148]. Under elevated CO_2_, reduced NR activity limits nitrogen assimilation and promotes reallocation away from photosynthetic machinery, resulting in decreased investment in Rubisco and light-harvesting proteins. Empirical studies in rice have demonstrated that photosynthetic acclimation to elevated CO_2_ involves marked adjustments in nitrogen allocation, with preferential reductions in Rubisco activity and chlorophyll content relative to other nitrogen pools [149]. In wheat, nitrogen limitation under elevated CO_2_ similarly redistributes nitrogen among photosynthetic components, effectively reducing the nitrogen investment per unit carbon gain [150].

Optimizing photosynthetic nitrogen-use efficiency (PNUE) under elevated CO_2_ requires dynamic nitrogen allocation among photosynthetic components. Studies show that acclimation involves reduced investment in Rubisco and other photosynthetic proteins, with nitrogen being reallocated to support growth and reproduction [151,152,153]. In tomato, nitrogen deficiency further decreases PNUE through reduced Rubisco allocation despite maintained chlorophyll levels [154]. Because elevated CO_2_ can constrain NR-mediated nitrogen assimilation, the resulting reduction in nitrogen availability limits investment in the photosynthetic apparatus and necessitates trade-offs between carboxylation and electron transport capacity [155,156]. Thus, post-translational regulation of NR acts as a key control point governing nitrogen flow into photosynthetic machinery and the extent of photosynthetic acclimation.

### 8.2. Reduced NR Activity, Decreased Amino Acid Synthesis, and Reduced Photosynthetic Capacity

Reduced NR activity restricts nitrate assimilation, limiting ammonium production and, in turn, amino acid synthesis via the GS/GOGAT pathway [12,126]. In tomato, nitrogen starvation downregulates NR- and GS-related genes, reduces enzyme activities, and disrupts amino acid metabolism, particularly glutamate, a key hub of nitrogen distribution [22,157]. Under elevated CO_2_, reduced photorespiration and nitrate assimilation further disturb carbon–nitrogen balance, impairing the synthesis of Rubisco and other Calvin cycle proteins required for sustained photosynthetic capacity. In tobacco, elevated CO_2_ suppresses NR activity and leaf protein content concurrently, establishing a direct correlation between nitrogen assimilation capacity and the maintenance of carboxylation efficiency [158,159].

Reduced amino acid synthesis limits the production of nitrogen-rich photosynthetic proteins, particularly Rubisco. Evidence from rice and tobacco shows that reduced nitrogen allocation or impaired NR activity suppresses Rubisco synthesis, leading to photosynthetic acclimation characterized by lower maximum carboxylation capacity [160]. Field studies using free-air CO_2_ enrichment (FACE) demonstrate that rice exhibits decreased Rubisco concentration and photosynthetic efficiency during later growth stages under elevated CO_2_, coincident with reduced nitrogen assimilation rates [161,162]. Ryegrass under low nitrogen nutrition shows marked decreases in Rubisco protein and carboxylation capacity when grown under elevated CO_2_, whereas high nitrogen supply mitigates these effects [163]. In tomato, optimal nitrogen supply maintains elevated NR and GS activities, supporting robust amino acid synthesis and sustaining photosynthetic capacity, whereas nitrogen excess or deficiency disrupts this balance, impairing electron transport efficiency and carbon fixation rates [27]. This biochemical limitation must nonetheless be distinguished from stomatal effects. Elevated CO_2_ generally reduces stomatal conductance, thereby lowering the CO_2_ diffusive limitation on photosynthesis in some cases, thereby improving water-use efficiency. Vega-Mas et al. [17] observed specific stomatal closure in 7.5 mM NH_4_^+^-fed tomato plants under elevated CO_2_ (800 ppm), which improved water-use efficiency but simultaneously compromised plant nitrogen status. This illustrates that stomatal and biochemical limitations can act in opposing directions: while elevated CO_2_ alleviates diffusive constraints on CO_2_ supply, the concurrent suppression of nitrogen assimilation mediated in part through NR inhibition imposes biochemical constraints on photosynthetic capacity that are independent of, and can ultimately override, the stomatal benefits [164,165]. Reduced photorespiration under elevated CO_2_ can further constrain nitrogen assimilation by limiting carbon skeleton availability and reducing support for nitrate reduction [10,166]. Consequently, under nitrogen limitation or decreased NR activity, photosynthetic acclimation becomes increasingly driven by biochemical constraints, as reduced amino acid supply restricts the synthesis of Rubisco and other Calvin cycle enzymes, leading to declines in both maximum carboxylation capacity (V_cmax_) and electron transport capacity (J_max_) [167].

### 8.3. Feedback Inhibition Due to Carbohydrate Accumulation

Elevated CO_2_-induced carbohydrate accumulation acts as a central feedback signal coordinating nitrogen assimilation and photosynthetic acclimation. Rather than simply reflecting enhanced carbon fixation, accumulated sugars regulate NR activity and photosynthetic gene expression through interconnected transcriptional and post-translational mechanisms, preventing excessive carbon assimilation when nitrogen availability or sink demand becomes limiting [103,168]. In tomato and other higher plants, sugar signals can override nitrogen-deficiency responses, while the reversible activation state of NR enables integration of fluctuating carbon availability over diurnal cycles [47].

Glutamine and asparagine act as feedback signals that repress NR expression and activity when carbon skeleton availability is limiting, thereby restricting nitrogen assimilation [137]. Research in tobacco and maize demonstrates that elevated tissue concentrations of glutamine correlate with reduced NR mRNA stability and protein accumulation, mediated through signaling pathways that sense the relative concentrations of glutamine versus 2-oxoglutarate [126,169]. Under elevated CO_2_, accelerated carbohydrate accumulation in tomato and other species exacerbates this feedback inhibition, contributing to photosynthetic acclimation as the C:N ratio increases and nitrogen assimilation becomes progressively constrained [170,171]. Collectively, these studies indicate that elevated CO_2_ promotes sugar accumulation and carbohydrate metabolic reprogramming, which function not only as metabolic consequences of enhanced photosynthesis but also as signaling mechanisms coordinating carbon and nitrogen metabolism. As soluble sugars accumulate, carbon demand becomes increasingly decoupled from nitrogen assimilation, resulting in feedback repression of NR and photosynthetic processes to restore carbon–nitrogen balance. Thus, carbohydrate signaling represents a major component of photosynthetic acclimation by integrating source–sink relationships with nitrogen availability rather than acting through independent metabolic pathways [130,131].

In common bean, high nitrogen supply exacerbates photosynthetic acclimation under elevated CO_2_ by increasing leaf area ratio and non-structural carbohydrate accumulation, resulting in coordinated repression of nitrate reductase (NR) and photosynthetic functions through sugar-sensing pathways [12,145]. Similarly, in rice, source–sink limitations during reproductive development reduce nitrogen uptake and assimilation, accelerating declines in photosynthetic capacity under elevated CO_2_ [161,172,173]. Together, these findings identify NR as a key integrator of carbon and nitrogen status, linking whole-plant source–sink balance to photosynthetic acclimation.

In addition to its metabolic role, NR also regulates nitric oxide (NO) production under elevated CO_2_, providing an important link between carbon–nitrogen metabolism and stomatal signaling. Although NR-derived NO promotes stomatal closure and improves water-use efficiency, current evidence suggests that this response is not the primary driver of photosynthetic acclimation. Instead, acclimation is predominantly governed by the biochemical limitation imposed by reduced NR activity, decreased nitrogen assimilation, and restricted synthesis of nitrogen-rich photosynthetic proteins. Nevertheless, prolonged NO-mediated stomatal responses may indirectly reinforce carbon–nitrogen imbalance by modifying source–sink relationships and nutrient acquisition, thereby complementing the biochemical mechanisms underlying photosynthetic acclimation.

## 9. Nitrate Reductase as a Hub Linking Nitrogen Metabolism and Signaling Under Elevated CO_2_

The responses of tomato (*Solanum lycopersicum*) to elevated CO_2_ (eCO_2_) are closely linked to nitrogen metabolism through nitrate reductase (NR) and its signaling product nitric oxide (NO). Elevated CO_2_ alters stomatal behavior, nitrogen assimilation, and stress responses, with NR acting as a key integrator of carbon and nitrogen status. Through its dual role in nitrate reduction and NO production, NR coordinates physiological, metabolic, and defense-related responses to elevated CO_2_. Beyond its function in nitrogen assimilation described in the previous section, NR-derived NO links metabolic regulation with stomatal signaling. While NO-mediated stomatal closure enhances water-use efficiency and stress adaptation, it primarily complements rather than drives photosynthetic acclimation, which is largely determined by biochemical constraints on nitrogen assimilation.

### 9.1. Nitrate Reductase in Whole-Plant Nitrogen Metabolism

Studies investigating tomato responses to (eCO_2_) typically utilize cultivated varieties such as *Solanum lycopersicum* L., often focusing on standard cultivars used in physiological assays [174]. Ambient CO_2_ concentrations are generally maintained at approximately 380–400 ppm, while elevated conditions are set at 700–800 ppm [175]. Under these elevated conditions, tomato plants exhibit enhanced photosynthetic rates and biomass accumulation, which fundamentally shifts the carbon-to-nitrogen (C:N) balance [176,177].

Elevated CO_2_ (eCO_2_) alters nitrate reductase (NR) activity in a nitrogen-dependent manner. Under adequate nitrate supply, eCO_2_ often suppresses NR activity and nitrogen assimilation through carbohydrate-mediated feedback, contributing to nitrogen dilution despite increased biomass, whereas under nitrogen limitation, it may enhance NR activity to improve nitrogen-use efficiency [178,179,180]. Elevated CO_2_ also promotes NO-mediated root growth and nutrient acquisition, but reduced NR activity can constrain amino acid synthesis and nitrogen assimilation. These responses are strongly influenced by source–sink balance, with excess carbohydrate accumulation reinforcing feedback inhibition of nitrogen metabolism when sink demand is insufficient [181,182].

### 9.2. NR-Derived Nitric Oxide Production

Nitric oxide (NO) acts as a pivotal signaling molecule in tomato responses to (eCO_2_), with NR identified as a primary enzymatic source of NO production under specific physiological conditions [183]. In tomato guard cells, (eCO_2_) triggers a rapid increase in endogenous NO levels, which is essential for inducing stomatal closure [184]. This NO accumulation is directly linked to NR activity, as demonstrated by NR inhibitors and NO scavengers, which abolish the eCO_2_-induced stomatal closure response [185]. Nitric oxide (NO) under elevated CO_2_ is commonly quantified using the fluorescent probe DAF-FM DA coupled with laser-scanning confocal microscopy to visualize NO accumulation in guard cells and roots [177]. Elevated CO_2_ enhances NR-dependent NO production, particularly in roots, where it promotes lateral root development. Although NOS-like activities may also contribute, NR remains the primary source of NO during nitrogen assimilation-related signaling [183,184]. Furthermore, NR silencing or inhibition markedly reduces NO accumulation, confirming its central role in elevated CO_2_-induced signaling pathways. Although NR-derived NO promotes stomatal closure under elevated CO_2_, available evidence indicates that this response mainly optimizes gas exchange and water conservation. The decline in photosynthetic capacity associated with acclimation is instead primarily attributable to reduced nitrogen assimilation and Rubisco synthesis, indicating that NO signaling functions as a complementary regulatory pathway rather than the principal cause of carbon–nitrogen imbalance.

### 9.3. Crosstalk Between NO, ROS, and Hormonal Signaling

The eCO_2_ response network in tomato involves crosstalk among nitric oxide (NO), reactive oxygen species (ROS), and ABA signaling. Elevated CO_2_ stimulates H_2_O_2_ and NO accumulation in guard cells, where ROS and NO act cooperatively to induce stomatal closure and enhance water-use efficiency [141,146,186,187,188]. ROS production, primarily via RBOH-dependent pathways, precedes or accompanies NO generation, activating downstream signaling components. Genetic regulation further modulates this response, as the transcription factor *SlWRKY81* negatively regulates drought tolerance by suppressing NO accumulation in guard cells [189]. ABA signaling components, such as *OST1* and *SLAC1*, are implicated in this pathway, in which NO and ROS activate anion channels, leading to membrane depolarization and potassium efflux [146,190]. While salicylic acid (SA) and jasmonic acid (JA) are involved in broader stress responses, the immediate stomatal response to eCO_2_ is primarily driven by the ABA-NO-ROS axis [191].

### 9.4. Defense Responses and Stress Signaling

Elevated CO_2_ enhances tomato defense against bacterial pathogens, such as *Pseudomonas syringae* pv. *tomato*, largely through stomatal closure, which restricts pathogen entry [174]. However, beyond physical barriers, NR-mediated NO production directly activates defense signaling pathways [192]. NO acts as a signaling molecule that induces the expression of pathogenesis-related (PR) genes and enhances the oxidative burst required for hypersensitive response-like defences [193]. In both compatible and incompatible interactions with pathogens, NO and ROS accumulate synergistically, modulating the defense response [194]. Under eCO_2_, the enhanced NO production via NR contributes to a primed defense state, allowing tomato plants to respond more robustly to biotic stress [195]. Additionally, NR-dependent NO signaling alleviates oxidative damage under abiotic stresses, such as low nitrogen or salinity, by upregulating antioxidant enzymes, including superoxide dismutase (SOD) and catalase (CAT), via MAPK signaling pathways [196,197,198]. This dual role of NR-derived NO in both stomatal regulation and defense activation underscores its importance in shaping plant resilience under changing atmospheric conditions [183,185].

## 10. Conclusions

Elevated atmospheric CO_2_ profoundly alters carbon–nitrogen interactions in tomato by enhancing carbon assimilation while frequently constraining nitrogen acquisition and assimilation. This review highlights nitrate reductase (NR) as the central metabolic and signaling hub linking nitrate reduction, carbon metabolism, nitric oxide (NO) signaling, and photosynthetic acclimation under elevated CO_2_. Current evidence demonstrates that elevated CO_2_ regulates NR through interconnected effects on photorespiration, carbohydrate-mediated feedback, redox balance, source–sink relationships, and nitrogen availability, resulting in tissue-specific responses and long-term metabolic acclimation. The review further summarizes the biochemical basis of nitrogen assimilation, the structural and functional biology of NR, and the multiple layers of NR regulation, including transcriptional, post-translational, and metabolic control. Evidence from physiological and multi-omics studies indicates that elevated CO_2_ reshapes carbon skeleton provision, amino acid biosynthesis, and nitrogen metabolism, while NR-dependent NO production integrates nitrogen metabolism with stomatal regulation through ABA-independent H_2_O_2_–NO signaling pathways. Collectively, these findings establish NR as a key coordinator of carbon–nitrogen homeostasis and plant adaptation to future climate conditions. Despite these advances, major knowledge gaps remain. Direct evidence for NR phosphorylation dynamics, 14-3-3 protein interactions, redox-mediated regulation, and tissue-specific post-translational control under elevated CO_2_ is still scarce. Likewise, comprehensive transcriptomic, proteomic, phosphoproteomic, metabolomic, and translatomic investigations specifically targeting NR regulation in tomato remain limited. Future research should integrate physiological, biochemical, and multi-omics approaches with gene-editing technologies and functional validation to unravel the molecular mechanisms governing NR regulation across different tissues and environmental conditions. Improving our understanding of NR-mediated carbon–nitrogen crosstalk will facilitate the development of tomato cultivars with enhanced nitrogen-use efficiency, sustained photosynthetic performance, and improved productivity under future elevated atmospheric CO_2_. Such advances will contribute to climate-resilient crop improvement and more sustainable agricultural production systems.

## Figures and Tables

**Table 1:** Omics studies investigating tomato responses to elevated CO_2_ and their implications for NR regulation.

Study	Tomato Genotype	CO_2_ Treatment	N Source	Tissue	Omics Approach
Van Oosten et al. [131]	Not specified	Ambient vs. elevated CO_2_	-	Leaves	Transcriptomics, metabolomics
Shi et al. [141]	Ailsa Craig, notabilis	380 vs. 800 ppm	-	Leaves	qPCR
Gray et al. [142]	M82, LA0716	400 vs. 700 ppm	6 mM NH_4_NO_3_	Leaves, roots	RNA-seq, TRAP-Seq, metabolomics
Vega-Mas et al. [17]	Agora Hybrid F1	400 vs. 800 ppm	NH_4_^+^, NO_3_^−^ (5–15 mM)	Whole plant	Metabolomics, proteomics
Jayawardena et al. [13]	Not specified	400 vs. 700 ppm	NH_4_^+^, NO_3_^−^	Roots	Proteomics
Vega-Mas et al. [139]	*S. lycopersicum* L.	400 vs. 800 ppm	NH_4_^+^, NO_3_^−^	Leaves	Transcriptomics
Zhou et al. [143]	SlMOT2-overexpressing tomato	Ambient CO_2_	Nitrate stress	Seedlings	RNA-seq
Desaint et al. [144]	Tomato introgression lines	Ambient CO_2_	Nitrogen deficiency	Roots, shoots	QTL mapping + RNA-seq

**Table 2:** Proposed mechanisms linking elevated CO_2_ to NR regulation and photosynthetic acclimation in tomato.

Mechanism	Effect of Elevated CO_2_	Consequence for NR	Physiological Outcome	References
Reduced photorespiration	Lower photorespiratory flux and reductant supply	Reduced nitrate assimilation	Lower amino acid synthesis and N-use efficiency	Searles & Bloom [16]; Bloom et al. [1]; Krämer et al. [10]
Sugar accumulation	Increased glucose/fructose signaling	Feedback repression of NR and photosynthetic genes	Photosynthetic acclimation	Van Oosten et al. [131]
Source–sink imbalance	Excess carbohydrate accumulation	Reduced NR activity and N assimilation	Nitrogen dilution	Yelle et al. [111]; Jifon & Wolfe [145]
Post-translational regulation	Phosphorylation and 14-3-3 binding	NR inactivation	Reduced nitrate reduction	Kaiser & Huber [23]; Lillo et al. [18]
Altered carbon skeleton metabolism	Changes in TCA, glyoxylate, and photorespiratory pathways	Modified N assimilation capacity	Amino acid reprogramming	Gray et al. [142]; Vega-Mas et al. [17,139]
NR-derived NO signaling	Activation of H_2_O_2_–NO pathway	NR-mediated signal transduction	Stomatal closure and improved WUE	Shi et al. [141]; García-Mata & Lamattina [146]
Nitrogen source effects	Differential responses under NH_4_^+^ and NO_3_^−^ nutrition	Distinct regulation of N assimilation pathways	Altered growth and acclimation responses	Vega-Mas et al. [15,17,139]; Jayawardena et al. [13]

## Data Availability

No new data were created or analyzed in this study. Data sharing is not applicable to this article.
